# Jiaweishaoyao Decoction Alleviates DSS-Induced Ulcerative Colitis via Inhibiting Inflammation

**DOI:** 10.1155/2020/7182874

**Published:** 2020-05-27

**Authors:** Huixia Qiao, Yahui Huang, Xiaoyan Chen, Long Yang, Yue Wang, Rongrong Xu

**Affiliations:** Department of Digestive, Xi'an Traditional Chinese Medicine Hospital, No. 69, Fengcheng Eight Road, Weiyang District, Xi'an City, Shaanxi Province 710021, China

## Abstract

**Purpose:**

Jiaweishaoyao decoction (JWSYD) is a traditional prescription of Chinese medicine that is initially used for the treatment of diarrhea. This study is aimed at investigating the effects of JWSYD on DSS-induced ulcerative colitis (UC).

**Methods:**

DSS-induced UC mice and LPS-induced RAW264.7 cells were used as the UC model *in vivo* and *in vitro*. UC was assessed by body weight, disease activity index (DAI), colon length, spleen weight, and histopathological score (HE staining). The levels of TNF-*α*, IL-1*β*, and IL-6 were analyzed by ELISA and qRT-PCR. The levels of NLRP3 inflammasome- and NF-*κ*B pathway-associated proteins were measured by western blot.

**Results:**

JWSYD alleviated DSS-induced UC in respect to body weight, DAI, colon length, spleen weight, and histopathological score. JWSYD reduced the levels of TNF-*α*, IL-1*β*, and IL-6 in DSS-induced UC mice and the supernatants of LPS-induced RAW264.7 cells. JWSYD suppressed the protein levels of inflammasome-associated proteins, including NLRP3, ASC1, Procaspase-1, Cleaved caspase-1, and Cleaved IL-1*β* in DSS-induced UC mice and LPS-induced RAW264.7 cells. In addition, JWSYD suppressed the NF-*κ*B pathway *in vitro* and *in vivo*.

**Conclusion:**

JWSYD alleviated DSS-induced UC via inhibiting the NLRP3 inflammasome and NF-*κ*B pathway.

## 1. Introduction

Ulcerative colitis (UC) is a common chronic inflammatory intestinal disease, which belongs to inflammatory bowel disease (IBD). Generally, pathogenic sites of UC are in the colon and rectum, and clinical symptoms of UC mainly include abdominal pain, diarrhea, and ulcer [1, 2]. UC is closely correlated with colorectal cancer (CRC). After UC is diagnosed for 8-10 years, the risk of CRC begins to rise [3]. The traditional treatment of UC is limited in long duration and side effects [4]. Therefore, it is necessary for us to seek an effective therapeutic method for UC.

Intestinal immune system plays a vital role in the secretion of inflammatory cytokines and the protection of the mucous layer and epithelial tissues [5]. UC is closely connected with the dysfunction of the intestinal immune system. As is well-known, NLRP3 inflammasome is mainly stimulated in macrophages that can generate cytokines [6]. Previous studies have revealed that the NLRP3 inflammasome participates in a variety of diseases, such as acute myocardial infarction and kidney and immune diseases [7–9]. The NLRP3 inflammasome may act as an effective target for diagnosing IBD [10]. Zhou et al. [11] revealed that the inhibition of NLRP3 inflammasome can alleviate DSS-induced UC. Meanwhile, the NF-*κ*B pathway has been considered to play a crucial role on inflammation of DSS-induced UC [12]. Shon et al. [13] testified that an inhibitor of the NF-*κ*B pathway decreases the severity of DSS-induced UC. Luo et al. [14] indicated that the NF-*κ*B pathway activates the NLRP3 inflammasome in alveolar macrophages. Li et al. [15] proved that CPT-11 activates the NLRP3 inflammasome through the NF-*κ*B pathway. To sum up, the NLRP3 inflammasome and NF-*κ*B pathway may be underlying targets for the treatment of UC.

Shaoyao decoction, containing *Radix Paeoniae Alba*, *Radix Angelicae Sinensis*, *Rhizoma Coptidis*, *Semen Arecae*, *Radix Aucklandiae*, *Radix Et Rhizoma Glycyrrhizae*, *Radix Et Rhizoma Rhei*, *Radix Scutellariae*, and *Cortex Cinnamomi* (dry weight ratio of 30 : 15 : 15 : 6 : 6 : 6:9 : 15 : 7.5), is a traditional Chinese medicine compound. A previous study concluded that Shaoyao decoction improves the response of anti-inflammation via the TLR4/NF-*κ*B pathway in UC [16]. A similar study has also demonstrated that Shaoyao decoction suppresses the secretion of inflammatory cytokines, thereby alleviating colitis-associated CRC [17]. Jiaweishaoyao decoction (JWSYD), containing *Radix Et Rhizoma Glycyrrhizae*, *Semen Arecae*, *Magnolia officinalis Rehd et Wils*, *Crataegus pinnatifida Bunge*, *Radix Et Rhizoma Rhei*, *Massa Medicata Fermentata*, *Radix Paeoniae Alba*, and *Radix Angelicae Sinensis* (dry weight ratio of 1 : 2 : 2 : 2:3 : 3 : 5 : 5), is a modified Shaoyao decoction. However, the regulatory effect and mechanism of JWSYD on UC remain clear.

In our study, we evaluated the function of JWSYD in DSS-induced UC mice and LPS-induced RAW264.7 cells. Our study may provide a novel target for UC treatment.

## 2. Methods

### 2.1. Experimental Animals

Male C57BL/6 mice of 6 weeks old (18-22 g) were purchased from the Animal Center of Medical Research Institute, Academy of Military Sciences (Beijing, China). The mice were fed freely in a SPF laboratory under a 12-hour cycle of light and dark. All experimental procedures were performed in accordance with animal management regulations of the Health Ministry of China and approved by the medical ethics committee of our hospital. After the study, all animals were anesthetized by intraperitoneal injection of sodium pentobarbital (80 mg/kg) and then sacrificed by cervical dislocation.

### 2.2. DSS-Induced UC Model

A UC model was established in mice by feeding 3% (*w*/*v*) DSS (MP Biomedicals, Santa Ana, USA) every 2 days for 1 week. Mice in the control group were given an equal volume of saline (*N* = 20). UC mice were then divided into two groups: DSS group and DSS+JWSYD group (*N* = 20 each group). UC mice in the DSS+JWSYD group were fed with JWSYD (22 g/kg) every day for 1 week. JWSYD was obtained from the Department of Traditional Chinese Medicine of our hospital, and the concentration was determined by our experimental experience (22 g/kg has no toxicity to mice). UC mice in the DSS group were fed with an equal volume of saline. The body weight, disease activity index (DAI), colon length, and spleen weight were recorded. Colon tissues were frozen in liquid nitrogen and used for subsequent experiments.

### 2.3. Histological Analysis

Colon tissues were fixed in 5% formalin, dehydrated and embedded in wax, and then cut at 5 *μ*m and stained with hematoxylin and eosin (HE). The sections were observed under an optional microscope (Olympus CKX53, Tokyo, Japan). Histopathological score was assessed according to the scoring system reported by Rachmilewitz et al. [18].

### 2.4. Cell Culture and Groups

Mouse mononuclear macrophages (RAW264.7 cells, #TCM13) were purchased from the Cell Center of Shanghai (Shanghai, China). RAW264.7 cells were cultured in RPMI-1640 medium (Gibco, Grand Island, USA) with 10% FBS (Gibco) and 1% streptomycin/penicillin (Gibco) at 37°C with 5% CO_2_. Cells were divided into three groups: mock group, LPS group, and JWSYD+LPS group. Cells in the LPS group were treated with LPS (2 *μ*g/ml) for 4 h. Cells in the JWSYD+LPS group were treated with LPS (2 *μ*g/ml) and JWSYD (8 mg/ml) for 4 h. Cells without treatment were considered the mock group.

### 2.5. Quantitative Real-Time PCR (qRT-PCR) Assay

Total RNA of tissues/cells was extracted by Trizol (Invitrogen, Carlsbad, USA). The cDNA was synthesized by a reverse-transcribed kit (Transgene, Beijing, China). Then, qRT-PCR was conducted by a SYBR RT-PCR kit (Takara, Beijing, China) on LightCycler® 480 (Roche, Basel, Switzerland) with the following conditions: an initial of 10 min at 95°C, followed by 40 cycles of 95°C for 10 s, 60°C for 20 s, and 72°C for 34 s. GAPDH was used as an internal control. The relative expression was calculated using the 2^−*ΔΔ*CT^ method. The primer sequences used in this study were as follows: IL-6 (forward): 5′-TACCACTTCACAAGTCGGAGGC-3′, IL-6 (reverse): 5′-CTGCAAGTGCATCATCGTTGTTC-3′; IL-1*β* (forward): 5′-TTGTTGATGTGCTGCTGTGA-3′, IL-1*β* (reverse): 5′-TGTGAAATGCCACCTTTTGA-3′; TNF-*α* (forward): 5′-GGTCTGGGCCATAGAACTGA-3′, TNF-*α* (reverse): 5′-CAGCCTCTTCTATTCCTGC-3′; and GAPDH (forward): 5′-AGGTCGGTGTGAACGGATTTG-3′, GAPDH (reverse): 5′-TGTAGACCATGTAGTTGAGTCA-3′.

### 2.6. Western Blot

Tissue and cell proteins were extracted by RIPA lysis buffer and separated by SDS-PAGE. Subsequently, the protein was transferred to PVDF membrane and incubated with a primary antibody (GAPDH, NLRP3, Cleaved caspase-1, Pro caspase-1, Cleaved IL-1*β*, p-I*κ*B*α*, I*κ*B*α*, p-NF-*κ*B p65, NF-*κ*B p65 (1 : 1000, CST, Boston, USA) and ASC1 (1 : 1000, Abcam, Cambridge, USA)) overnight at 4°C. The membrane was then incubated with horseradish peroxidase-conjugated secondary antibody (anti-rabbit IgG, 1 : 10000, Sigma, St. Louis, USA) for 1 h. Finally, the protein bands were detected by an ECL kit and quantized using Quantity One 1-D Analysis Software (Bio-Rad, Hercules, USA).

### 2.7. ELISA

The concentrations of TNF-*α*, IL-1*β*, and IL-6 in serum and cell supernatants were detected by using specific ELISA kits (Thermo Fisher). The absorbance was measured by a microplate reader (Epoch 2, BioTek, Vermont, USA).

### 2.8. Statistical Analysis

GraphPad Prism 6 software (La Jolla, USA) and SPSS 22.0 Statistical Software (Chicago, USA) were adopted for statistical analysis. Data were presented as mean ± standard deviation. The differences between two groups were analyzed by Student's *t*-test. The differences among multiple groups were determined by one-way ANOVA followed by Tukey's post hoc test. All data were repeated three times. *P* < 0.05 was considered to be significantly different.

## 3. Results

### 3.1. JWSYD Improves DSS-Induced UC

**Figure 1:**
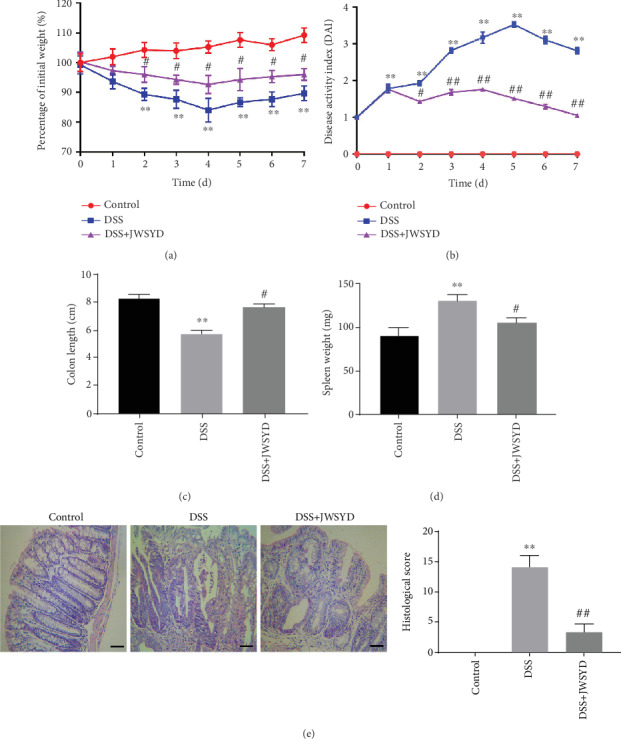
Jiaweishaoyao decoction (JWSYD) improved the DSS-induced ulcerative colitis (UC) in mice. (a) The percentage of initial weight from 0 to 7 days. (b) Disease activity index (DAI) from 0 to 7 days. (c) Colon length. (d) Spleen weight. (e) HE staining and histopathological score (magnification ×400). Bar = 100 *μ*m. ^∗∗^*P* < 0.01 compared with the control group; ^#^*P* < 0.05, ^##^*P* < 0.01 compared with the DSS group.

The effect of JWSYD on DSS-induced UC was evaluated in mice. The weight was decreased, and DAI score was increased in the DSS group compared with the control group (*P* < 0.01). After being treated with JWSYD, the weight was obviously increased and DAI score was decreased (*P* < 0.05) (Figures 1(a) and 1(b)). Meanwhile, the length of the colon was shorter and the weight of the spleen was greater in the DSS group than in the control group (*P* < 0.01). JWSYD had an obviously protective impact on colon shortening and splenomegaly (*P* < 0.05) (Figures 1(c) and 1(d)). As shown in Figure 1(e), HE revealed that the histopathological score of the DSS group was significantly increased compared with that of the control group, while JWSYD reduced the histopathological score obviously (*P* < 0.05). The above data indicated that JWSYD could protect DSS-induced UC.

### 3.2. JWSYD Reduces the Levels of Inflammatory Cytokines in DSS-Induced UC

**Figure 2:**
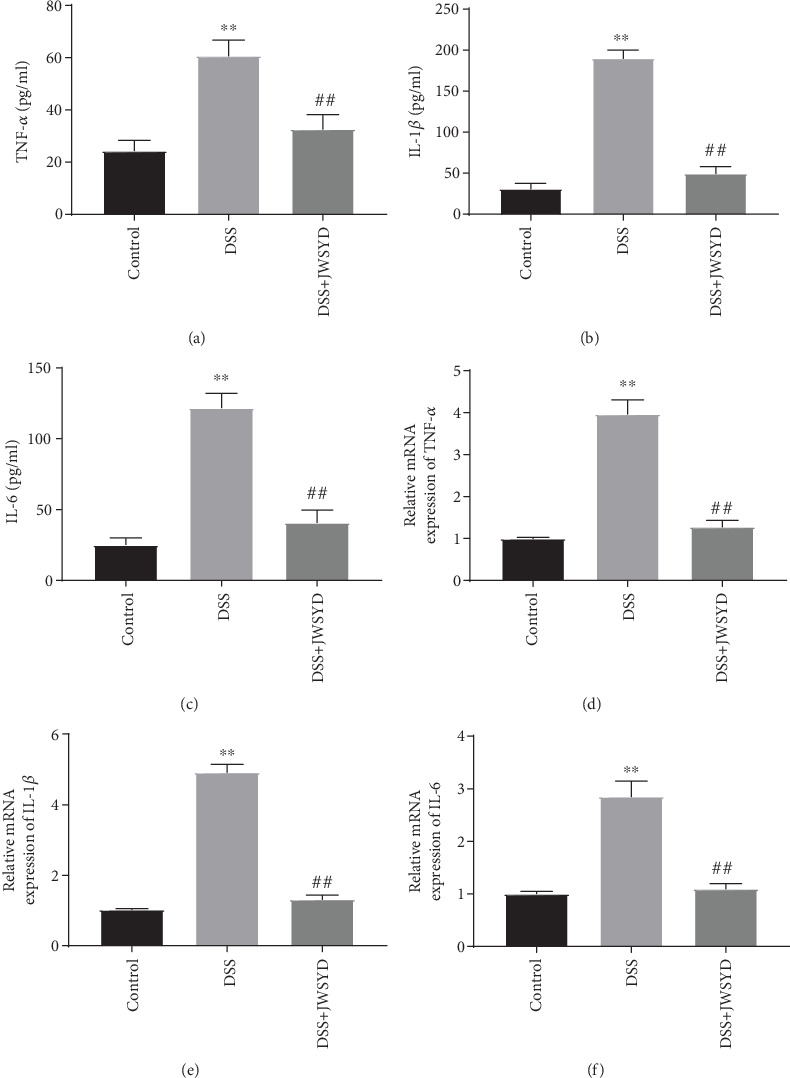
Jiaweishaoyao decoction (JWSYD) reduced the levels of inflammatory cytokines in DSS-induced ulcerative colitis (UC). (a–c) The levels of TNF-*α*, IL-1*β*, and IL-6 in serum of mice were detected by ELISA. (d–f) The mRNA levels of TNF-*α*, IL-1*β*, and IL-6 in colon tissues of mice were detected by qRT-PCR. ^∗∗^*P* < 0.01 compared with the control group; ^##^*P* < 0.01 compared with the DSS group.

The levels of inflammatory cytokines in the serum of mice were detected by ELISA. As shown in Figures 2(a)–2(c), the levels of TNF-*α*, IL-1*β*, and IL-6 in the DSS group were significantly higher than those in the control group (*P* < 0.01). The levels of TNF-*α*, IL-1*β*, and IL-6 in the DSS+JWSYD group were significantly lower than those in the DSS group (*P* < 0.01). We further explored the mRNA levels of TNF-*α*, IL-1*β*, and IL-6 in colon tissues using qRT-PCR. Consistent with the data of serum levels, the mRNAs levels of TNF-*α*, IL-1*β*, and IL-6 in the DSS+JWSYD group were dramatically declined compared with those in the DSS group (*P* < 0.01) (Figures 2(d)–2(f)). These results indicated that JWSYD could reduce the levels of inflammatory cytokines in DSS-induced UC.

### 3.3. JWSYD Inhibits NLRP3 Inflammasome in DSS-Induced UC

**Figure 3:**
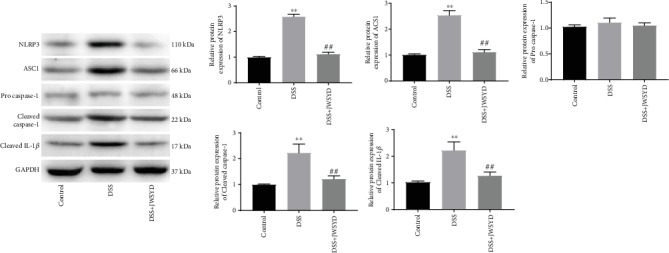
Jiaweishaoyao decoction (JWSYD) inhibited the NLRP3 inflammasome in DSS-induced ulcerative colitis (UC). The protein levels of NLRP3 inflammasome-associated proteins (NLRP3, ASC1, Procaspase-1, Cleaved caspase-1, and Cleaved IL-1*β*) were detected by western blot. ^∗∗^*P* < 0.01 compared with the control group; ^##^*P* < 0.01 compared with the DSS group.

The expression of NLRP3 inflammasome-associated proteins was detected by western blot. The protein levels of NLRP3, ASC1, Procaspase-1, Cleaved caspase-1, and Cleaved IL-1*β* were remarkably increased in the DSS group (*P* < 0.01). JWSYD decreased the levels of the above proteins compared with those in the DSS group (*P* < 0.01) (Figure 3). These data indicated that JWSYD could inhibit the NLRP3 inflammasome in DSS-induced UC.

### 3.4. JWSYD Inhibits the Inflammatory Reaction in LPS-Induced RAW264.7 Cells

**Figure 4:**
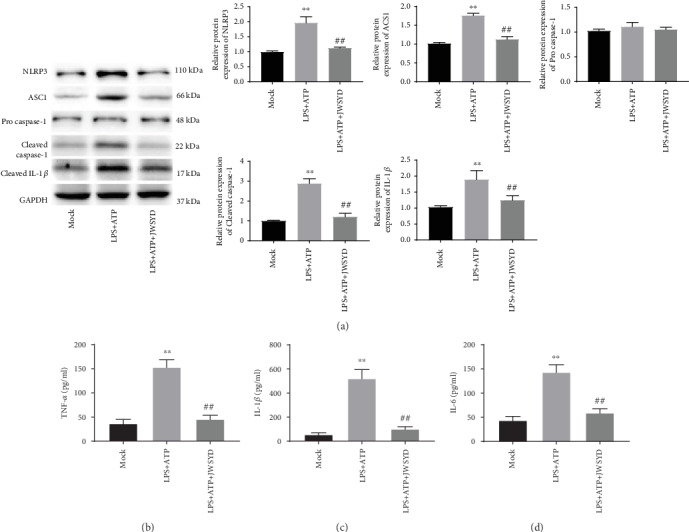
Jiaweishaoyao decoction (JWSYD) inhibited the inflammatory reaction in LPS-induced RAW264.7 cells. (a) Protein levels of NLRP3 inflammasome-associated proteins (NLRP3, ASC1, Procaspase-1, Cleaved caspase-1, and Cleaved IL-1*β*) in LPS-induced RAW264.7 cells. (b–d) The levels of TNF-*α*, IL-1*β*, and IL-6 were detected by ELISA in RAW264.7 cell supernatants. ^∗∗^*P* < 0.01 compared with the mock group; ^##^*P* < 0.01 compared with the LPS+ATP group.

We selected LPS-induced RAW264.7 cells to further explore the impact of JWSYD *in vitro*. Compared with the mock group, relative protein levels of NLRP3, ASC1, Cleaved caspase-1, Cleaved IL-1*β*, and Procaspase-1 were notably elevated in the LPS+ATP group (*P* < 0.01). JWSYD significantly decreased the protein levels of the above factors in the LPS+ATP+JWSYD group compared with the LPS+ATP group (*P* < 0.01) (Figure 4(a)). Similarly, ELISA results showed that JWSYD significantly reduced LPS-induced secretion of TNF-*α*, IL-1*β*, and IL-6 in RAW264.7 cell supernatants (*P* < 0.01) (Figures 4(b)–4(d)).

### 3.5. JWSYD Suppresses the NF-*κ*B Pathway

**Figure 5:**
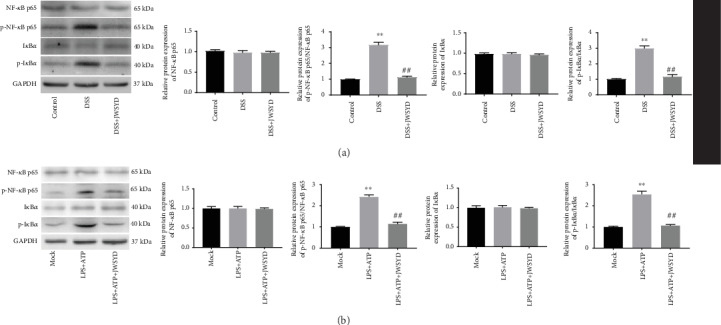
Jiaweishaoyao decoction (JWSYD) suppressed the NF-*κ*B pathway. (a) Protein levels of NF-*κ*B pathway-associated proteins (NF-*κ*B p65, I*κ*B*α*, p-NF-*κ*B p65, and p-I*κ*B*α*) were detected by western blot in colon tissues. ^∗∗^*P* < 0.01 compared with the control group; ^##^*P* < 0.01 compared with the DSS group. (b) Protein levels of NF-*κ*B pathway-associated proteins (NF-*κ*B p65, I*κ*B*α*, p-NF-*κ*B p65, and p-I*κ*B*α*) were detected by western blot in LPS-induced RAW264.7 cells. ^∗∗^*P* < 0.01 compared with the mock group; ^##^*P* < 0.01 compared with the LPS+ATP group.

Western blot showed that the protein levels of p-NF-*κ*B p65 and p-I*κ*B*α* in the DSS group were higher than those in the control group (*P* < 0.01). The protein levels of p-NF-*κ*B p65 and p-I*κ*B*α* in the DSS+JWSYD group were obviously decreased compared with those in the DSS group (*P* < 0.01). There was no significant difference in the protein levels of NF-*κ*B p65 and I*κ*B*α* in each group (*P* > 0.05) (Figure 5(a)). Similarly, the protein levels of p-NF-*κ*B p65 and p-I*κ*B*α* were increased obviously in the LPS+ATP group compared with those in the mock group. JWSYD decreased LPS-induced upregulation of p-NF-*κ*B p65 and p-I*κ*B*α* in the LPS+ATP+JWSYD group compared with the LPS+ATP group (*P* < 0.01) (Figure 5(b)). The above data indicated that JWSYD could suppress the NF-*κ*B pathway in DSS-induced UC and LPS-stimulated RAW264.7 cells.

## 4. Discussion

UC is known as a chronic disease with typical inflammatory imbalance in colon tissues [19]. In the current study, we established the model of DSS-induced UC mice *in vivo* and the model of LPS-induced RAW264.7 cells *in vitro*. We found that JWSYD alleviated DSS-induced UC in mice. Meanwhile, JWSYD reduced the levels of inflammatory cytokines (TNF-*α*, IL-1*β*, and IL-6) and the protein levels of inflammasome-associated proteins (NLRP3, ASC1, Procaspase-1, Cleaved caspase-1, and Cleaved IL-1*β*) in DSS-induced UC mice and LPS-induced RAW264.7 cells. Furthermore, JWSYD suppressed the NF-*κ*B pathway *in vitro* and *in vivo*.

Traditional Chinese medicine is widely used in the treatment of UC. For instance, paeoniflorin increases body weight, improves colon shortening, and reduces colon histology score by suppressing the expression of TLR4/NF-*κ*B/MAPK pathways [20]. Jeong et al. [21] concluded that the mixture of Rhizome and Anemarrhena can alleviate colitis. In our study, JWSYD mainly consist of paeoniae and rhizoma alleviated DSS-induced UC in respect to body weight, DAI, colon length, spleen weight, and histopathological score. Our results indicate that JWSYD can improve DSS-induced UC in mice. Since paeoniae and rhizome are key components of JWSYD, these two agents may contribute to the therapeutic effect of JWSYD on DSS-induced UC.

Increasing evidence has manifested that proinflammatory cytokines play a vital role in DSS-induced UC, which consist of TNF-*α*, IL-1*β*, and IL-6 [22]. TNF-*α* has been considered to be a vital cell factor in the aspect of mucosal damaged in IBD, and inhibition of TNF-*α* can be used as an effective therapy in IBD [23]. IL-1*β* and IL-6 are secreted from macrophages [24]. IL-1*β* is a target in the early stage of colitis [25]. IL-6 level of serum is associated with disease activity in IBD [26]. In our study, JWSYD markedly decreased the levels of TNF-*α*, IL-1*β*, and IL-6 in DSS-induced UC mice and LPS-induced RAW264.7 cell supernatants. Our results indicate that JWSYD can alleviate the production of inflammatory cytokines *in vivo* and *in vitro*. The anti-inflammatory effect of JWSYD is similar to that of some other traditional Chinese medicines as reported previously. For example, the modified Sanhuang decoction (*Rhizoma Coptidis*, *Radix Scutellariae*, *Cortex Phellodendri*, *Forsythia suspense*, *Moutan Cortex*, *Gardenia jasminoides Ellis*, *Radix Paeoniae Rubra*, and *Herba Menthae*; dry weight ratio of 1.5 : 3 : 3 : 4.5 : 6 : 4.5 : 3 : 3) decreases the expression of TNF-*α*, IL-1*β*, and IL-6 and improves DSS-induced UC [27]. Shaoyao decoction decreases the expression of TNF-*α*, IL-1*β*, and IL-6 in DSS-induced colitis-associated CRC [17].

The NLRP3 inflammasome is a protein complex including NLRP3, ASC, and Procaspase-1 [28]. The NLRP3 inflammasome, which can mediate the secretion of IL-1*β*, is activated in IBD [29, 30]. A previous research has stated that MCC950, an inhibitor of the NLRP3 inflammasome, suppresses the inflammation in inflammatory diseases [31]. Curcumin restrains the NLRP3 inflammasome to relieve the secretion of inflammatory cytokines in DSS-induced UC [32]. Likewise, Huaier extract inhibits the NLRP3 inflammasome and protects against DSS-induced UC in mice [33]. In our study, JWSYD decreased the protein levels of inflammasome-associated proteins, including NLRP3, ASC1, Procaspase-1, Cleaved caspase-1, and Cleaved IL-1*β* in DSS-induced UC mice and LPS-induced RAW264.7 cells. The results above indicate that JWSYD can suppress NLRP3 inflammasome in DSS-induced UC. The inhibition of NLRP3 inflammasome contributes to the remission of inflammation in UC.

NF-*κ*B is recognized as a transcription factor that exhibits a vital function in the immune system [34]. NF-*κ*B is an important point in the course of IBD, and the expression of p-NF-*κ*B is upregulated in DSS-induced UC [35, 36]. In addition, NF-*κ*B regulates the expression of NLRP3 and it is recognized as the first signal to reflect the activation state of the NLRP3 inflammasome [37]. In our study, JWSYD inhibited the expression of p-NF-*κ*B p65 and p-I*κ*B*α* in DSS-induced UC and LPS-induced RAW264.7 cells, suggesting that JWSYD suppressed the NF-*κ*B pathway. The inhibitory effect of JWSYD on the NF-*κ*B pathway in UC was similar to that of some other traditional Chinese medicines as reported previously. For examples, Shaoyao decoction improves the anti-inflammation response via the TLR4/NF-*κ*B pathway in UC [16]. Wogonoside protects against DSS-induced UC in mice by inhibiting the NF-*κ*B pathway [38]. To sum up, we suspect that JWSYD may relieve DSS-induced UC by blocking the NF-*κ*B pathway.

## 5. Conclusions

In conclusion, JWSYD improved DSS-induced UC in mice, probably by reducing inflammatory cytokines and inhibiting the NLRP3 inflammasome. The treatment effects of JWSYD on DSS-induced UC were associated with the blocking of the NF-*κ*B pathway. Our research provides new insights into the treatment of UC in the future.

## Data Availability

All data are available through the responsible corresponding author.
